# Supplementary material for: Genome-Wide Identification, Genomic Organization, and Characterization of Potassium Transport-Related Genes in Cajanus cajan and Their Role in Abiotic Stress
Source: Plants (Basel). 2021 Oct 20;10(11):2238. doi: 10.3390/plants10112238 (PMC8619154; doi:10.3390/plants10112238)
Supplement: Supplementary file 1 [file plants-10-02238-s001.zip › plants-1377469-supplementary.pdf]

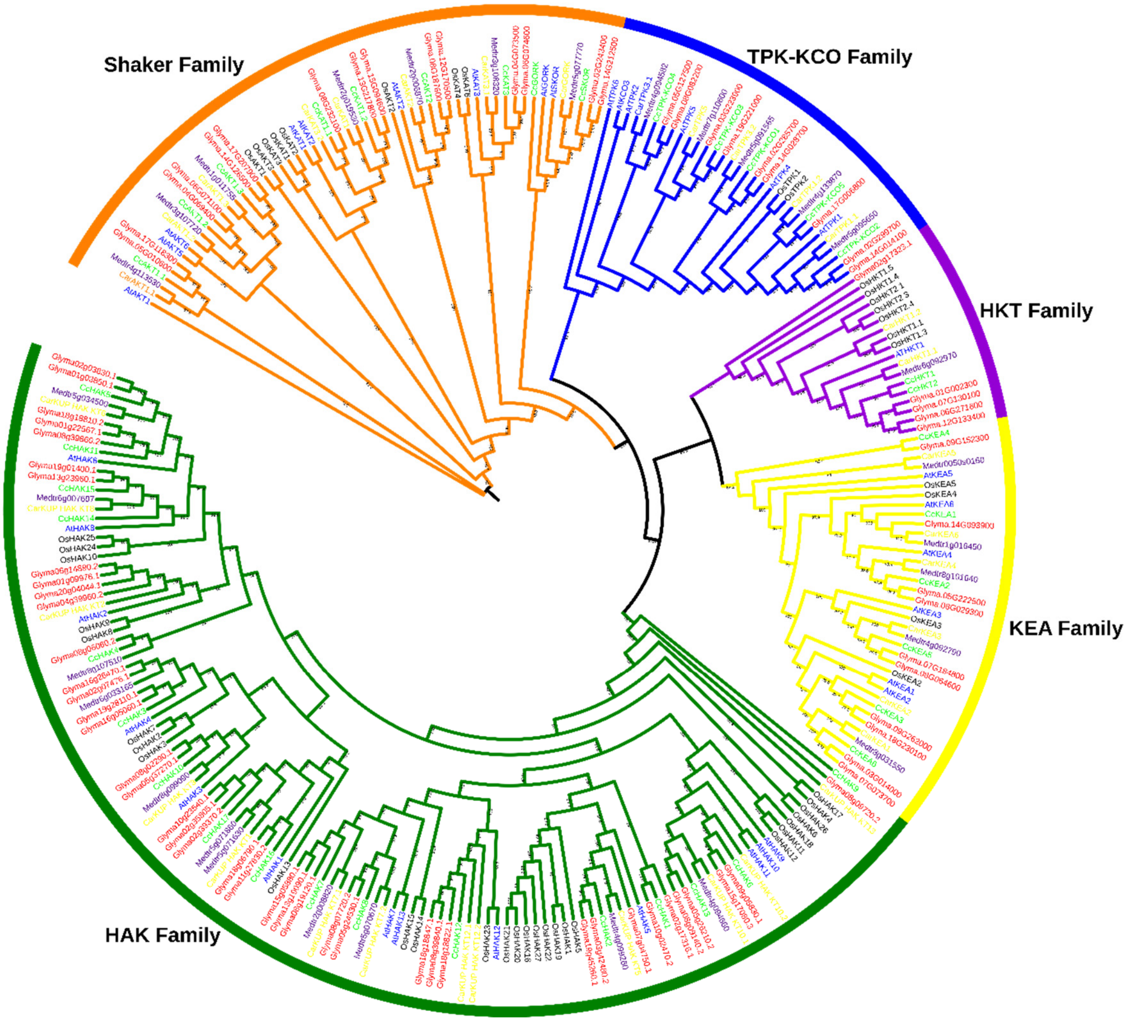

Figure S1. The evolutionary history was inferred by using the Maximum Likelihood method based on the JTT matrix-based model tree with the highest log likelihood (-119897.9497) is shown. Initial tree(s) for the heuristic search were obtained automatically by applying Neighbor-Join and BioNJ algorithms to a matrix of pairwise distances estimated using a JTT model, and then selecting the topology with superior log likelihood value. The tree is drawn to scale, with branch lengths measured in the number of substitutions per site. Evolutionary analyses were conducted in MEGAX. Visualization of phylogenetic tree was carried out through iTOL webserver.
